# Gradual increase in energy intake over 8 weeks with voluntary wheel running limits body weight change in male rats

**DOI:** 10.1017/S0007114525000194

**Published:** 2025-02-28

**Authors:** Isabelle Durocher, Daniel S. Grant, Marc R. Bomhof

**Affiliations:** Department of Kinesiology and Physical Education, University of Lethbridge, 4401 University Dr. W., Lethbridge, AB T1K 3M4, Canada

**Keywords:** Appetite-regulating hormones, Body weight, Energy compensation, Exercise, Wheel running

## Abstract

The influence of appetite and energy intake (EI) on energy compensation in response to chronic exercise remains poorly understood. This study examined the temporal impact of habitual exercise on EI and the homeostatic appetite regulators that influence energy compensation. Twelve-week-old male Sprague Dawley rats (*n* 30) fed an AIN-93M diet were randomised into one of three groups: (1) sedentary control (SED); (2) voluntary wheel exercise (EX) and (3) sedentary, weight-matched to aerobic exercise (SED-WM) for 8 weeks. Measures of EI, body weight and adiposity were assessed. Appetite-regulating hormones acyl ghrelin, active glucagon-like peptide (GLP)-1, leptin and insulin were measured in response to an oral glucose tolerance test. Rats with running wheels completed an average of 192 km over 8 weeks. While EI was initially reduced in EX, EI gradually increased with exercise training after week 1 (*P* < 0·05). Body weight was lower in EX relative to SED from weeks 3 to 5 but did not differ at the end of the study. Fat mass and long-term satiety hormones leptin and insulin were lower in EX (*P* < 0·05). No differences were observed in concentrations of the satiety hormone active GLP-1 or the orexigenic hormone acyl ghrelin. Short-term homeostatic regulators of appetite do not appear to be altered with exercise training. The reduction in adiposity and associated decrease in tonic satiety hormones leptin and insulin are likely contributors to the coupling of energy expenditure with EI over time with voluntary exercise.

Exercise, often pursued for weight management, is not always effective for sustained reductions in body weight^(1,2)^. Various defence mechanisms are engaged in response to energy expenditure from physical activity or diet-induced energy deficit^(3,4)^. Protection against sustained energy deficiency and weight reduction is achieved through a variety of mechanisms including alterations in resting energy expenditure^(5)^, reductions in non-exercise activity thermogenesis^(6–9)^, enhanced mechanical efficiency^(10,11)^ and increased energy intake (EI)^(12)^.

Acute and chronic exercise exerts divergent effects on the appetite-regulatory system and EI. An acute exercise bout generally induces short-term anorectic effects through transient changes in gut-derived, appetite-regulating hormones^(13)^. *Ad libitum* meals following an acute bout of exercise show limited increases in EI^(14,15)^. With repeated, regular bouts of exercise, completed over a 1–2-week period, a higher degree of energy compensation through increased EI is observed^(16–18)^. As exercise interventions increase in duration, the degree of energy compensation appears to increase^(6,12)^. Studies show that prolonged exercise over a 3-month period contributes to increased hunger, prospective food consumption and cravings for sweet foods^(19,20)^. With human research, it remains challenging to discern the degree of energy compensation through increased EI given the difficulty of obtaining precise measures of EI over an extended period of time^(21)^. Animal studies permit more accurate measurements of EI. Numerous rodent studies report elevated EI and no change in body weight when animals are provided with access to voluntary running wheels^(7,8,22,23)^. In contrast, many animal studies report an incomplete energy compensation and an overall lowering of body weight with prolonged exercise training using wheel running or a forced treadmill^(24–29)^.

The mechanisms by which prolonged exercise impacts appetite and EI are complex and remain unclear^(30)^. The homeostatic appetite-regulatory system involves constant communication between the gastrointestinal system, adipose tissue and appetite-regulating regions within the brain^(31,32)^. Anorexigenic hormones glucagon-like peptide-1 (GLP-1) and peptide YY, as well as the orexigenic hormone ghrelin, released from enteroendocrine cells within the gastrointestinal tract, communicate short-term energy status. In contrast, the anorexigenic hormones leptin and insulin, which fluctuate in a manner that is proportional to adipose tissue, signal long-term energy status^(32)^. Prolonged exercise studies in humans and animals have demonstrated increased concentrations of satiety hormone peptide YY^(23,33,34)^, late postprandial increases in the release of GLP-1^(35,36)^ and suppressed secretion of acylated ghrelin^(23,37)^. These satiety-inducing effects with chronic exercise are in contrast to the reductions in the short-term anorexigenic hormones that are observed with diet-induced, body weight reduction^(38)^. With exercise training and associated improvement in body composition, leptin and insulin are generally reduced^(26,29,36,39,40)^, an effect that is likely to increase hunger and energy compensation.

To further examine the relationship between anorexigenic and orexigenic appetite signalling with habitual exercise, this study assessed the temporal relationship between exercise, EI and homeostatic regulators of appetite over an 8-week period in male Sprague Dawley rats. A weight-matched, sedentary group was included to examine the weight-independent effects of exercise on appetite-regulating hormones.

## Materials and methods

### Animals and experimental protocol

This study was approved by the Animal Welfare Committee at the University of Lethbridge (Protocol #2104, approved March 30, 2021) and was completed in accordance with the ethical standards outlined in the Canadian Council on Animal Care. Thirty, 12-week-old male Sprague Dawley rats were obtained from the Charles Rivers Laboratories (Charles River, St. Constant, PQ). Animals were individually housed in techniplast cages with solid floors, shredded paper towels, crinkle paper bedding and polyvinyl chloride tubes. The rats were housed in temperature-controlled (20–22°C required) and humidity-controlled rooms with 12-hour light–dark cycles (07.00–19.00). Cages were cleaned weekly. All animals were monitored daily for pain, distress and discomfort.

Prior to randomisation, rats underwent a 7-d acclimatisation period with *ad libitum* access to water and an AIN-93M purified diet (Dyets Inc., Bethlehem, PA). The AIN-93M diet has uniform energy density and conforms to the recommendations set forth by the American Institute of Nutrition^(41)^. Rats were weighed daily throughout this period, and food intake was monitored. The nutrient breakdown from the AIN-93M diet is provided in Table 1.

**Table 1.** Composition of AIN-93M diet

Composition	AIN-93M
Casein (g/kg)	140
L-Cystine (g/kg)	1·8
Sucrose (g/kg)	100
Dyetrose (g/kg)	155
Maize starch (g/kg)	465·7
Soyabean oil (g/kg)	40
t-Butylhydroquinone (g/kg)	0·008
Cellulose (g/kg)	50
Mineral mix (g/kg)	35
Vitamin mix (g/kg)	10
Choline bitartrate (g/kg)	2·3
Energy (kcal/g)	3·6
Protein (% energy)	14·1
Carbohydrate (% energy)	76
Fat (% energy)	9·9

**Figure 1.**
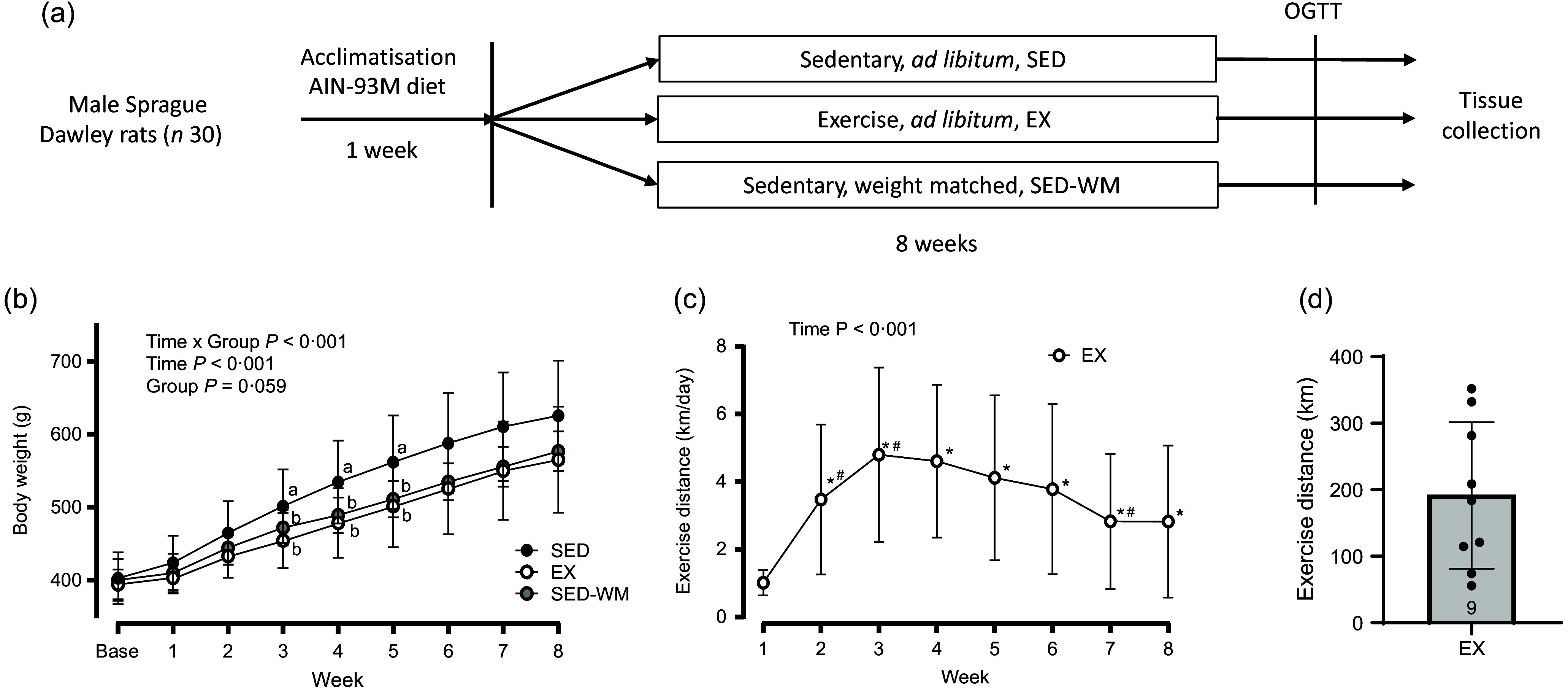
Schematic of study protocol (a). Weekly body weight for SED, EX and SED-WM (b), exercise distance for EX (c) and total exercise distance for EX (d) over 8 weeks. Values are means (sd). *n* 10 for SED and SED-WM; *n* 9 for EX. ^ab^Superscripts indicate significant group differences, labelled means at a time without a common letter differ, *P* < 0·05.*Significant difference from baseline, ^#^Significant difference from the week prior, *P* < 0·05. EX, exercise; GLP-1, glucagon-like peptide-1; OGTT, oral glucose tolerance test; SED, sedentary; SED-WM, sedentary - weight matched.

Sprague Dawley rats (*n* 30) were randomised into one of three groups: (1) sedentary control (SED); (2) voluntary aerobic exercise (EX) or (3) sedentary and weight-matched to aerobic exercise (SED-WM) for 8 weeks (*n* 10 rats/group). A stratified randomisation process was used to ensure equal average body weight between groups at the initiation of the study. The SED-WM group was included as a means to isolate the weight-independent effects of exercise-induced energy expenditure on appetite-regulating hormones. An overview of the study is provided in Fig 1(a).

### Exercise distance

Rats in EX had access to voluntary running wheels (Techniplast, Philadelphia, USA). Activity tracking (running distance) was monitored daily with Cateye Velo 9 cycling computers (Cateye Co. LTD, Osaka, Japan). The cycling computers were calibrated according to the circumference of the running wheels, which was measured at 96 cm. Rats were assessed daily for paw abrasions from the running wheels.

### Energy intake and diet manipulation

EI and body weight were precisely measured and recorded daily. Daily EI (3·6 kcal/g of food) was determined by subtracting the remaining food from the total food given from the day prior. SED and EX groups received food *ad libitum*. Food provisions for SED-WM were adjusted accordingly to ensure equal weight between EX and SED-WM. Based on previous research with exercise training, it was estimated that rats in SED-WM would require ∼80 % of the EI of SED^(42)^.

### Oral glucose tolerance test

One week prior to euthanasia, an oral glucose tolerance test (OGTT) was completed after an overnight fast and 12 h with the running wheels locked. Blood was sampled by placing an indwelling butterfly catheter in the lateral tail vein. Rats were placed on a heating pad to increase tail vein dilation. After being placed in a plastic tube restraint, the lateral tail vein was identified, and the sampling area was wiped with chlorhexidine antiseptic solution. A butterfly catheter was inserted into the tail vein and secured using tape. A 300-ul baseline blood sample was collected. After the baseline blood draw, rats were gavaged with an oral D-glucose (2 mg/kg BW) solution. Additional serial 300 ul blood collections were completed at the time points of 15, 30, 60, 90 and 120 min. Patency of the catheter was maintained through injection of 0·1 ml of heparin after all blood draws. An equal number of rats were assessed from each group for every day of OGTT testing. Collected blood was immediately transferred into cooled ethylenediaminetetraacetic acid spray-coated microcentrifuge tubes (BD, Mississauga, ON, Canada) containing diprotinin-A (10 μl/ml of blood; MilliporeSigma Corp., Burlington, MA, USA), sigma protease inhibitor (1 mg/ml of blood; SigmaFast, MilliporeSigma Corp) and Roche Pefabloc (1 mg/ml of blood; MilliporeSigma Corp) to prevent degradation of the hormones of interest. After each blood draw, blood glucose was measured using a OneTouch Glucose Meter (OneTouch, Ultra 2 Glucose Meter, Lifescan Inc., Malvern, PA, USA). Blood samples were centrifuged at 2000 rpm for 10 min at 4°C, and aliquoted plasma was stored at −80°C until analysis.

### Tissue and plasma collection

Following a 12-h overnight fast and a period of 12 h of locked wheels, rats were over-anaesthetised in an induction chamber with 5 % isoflurane. Once rats were in a state of deep anaesthesia, rats were switched to a nose cone with 2 % isoflurane. After an abdominal incision, the portal vein was identified and a 3-ml portal vein blood sample was collected and immediately mixed with protease inhibitors. Following a cardiac cut and heart removal, the retroperitoneal, peritoneal, epidydimal, inguinal and brown fat pads were precisely excised and weighed. Relative fat mass was calculated by summing the five fat pads and dividing by the end body weight.

### Biochemical analysis of appetite-regulating hormones

All appetite-regulating hormones, acylated ghrelin (active), glucagon-like peptide-1 (active), insulin and leptin were analysed using commercially available enzyme-linked immunosorbent assay (ELISA) kits (MilliporeSigma Corp, Burlington, MA, USA) according to the manufacturer’s directions. Corticosterone concentration was determined using corticosterone ELISA Kit (Arbor Assays, Ann Arbor, MI, USA). All blood samples from the OGTT were assayed in singles, as according to the National Centre for the Replacement, Refinement, & Reduction of Animals in Research, less than 2 ml of blood can be safely collected over 24 h^(43)^. Homeostatic model assessment of insulin resistance was calculated as (fasting glucose (mmol/l) × fasting inulin (mIU/ml))/22·5. The composite insulin sensitivity index was calculated using the formula: 10 000 \ √ ((Glucose, 0 min × Insulin, 0 min) × (Glucose, mean × Insulin, mean))^(44)^. A higher composite insulin sensitivity index score indicates improved insulin sensitivity.

### Statistical analysis

IBM SPSS 28.0 software was used to analyse data. To assess the normality of data, the Shapiro–Wilk test was used with *P* < 0·05, and Shapiro–Wilk < 0·8 statistics defined as significantly skewed. The appropriate transformation was used for data that were not normally distributed. To determine the difference for all data involving serial measures, a mixed-design ANOVA was used to assess time as the within-condition variable and group as the between-condition variable. A one-way ANOVA was used to determine differences between the treatment groups and time if a significant interaction was identified. A *post hoc* Tukey’s test was used to determine differences between treatment groups. Effect size (*d*) for post hoc pairwise comparisons was calculated using Cohen’s *d* (small = 0·20, medium = 0·50, large = 0·80). Effect size for repeated measures ANOVA was determined using partial eta squared (*η*_*p*_^*2*^ *)* (small = 0·01, medium = 0·06, large = 0·14). AUC measures were calculated by trapezoidal sums. Pearson correlation or Spearman correlation was used to assess the correlation between outcome variables. Sample size calculations were calculated using G * Power 3.1.9.4 with an *α* level of 0·05 and power level of 0·80. Changes in appetite-regulating hormones in previous studies utilising exercise training have ranged between 20 and 150 %^(23,45)^. Using standard deviation values (∼16 %) from previously conducted appetite-regulating hormone analysis in our laboratory, we estimated that a sample size of 10 rats per group was needed to detect a 20 % change in hormones of interest. For all statistical tests, *P* < 0·05 was considered statistically significant. All data are represented as the mean (sd).

## Results

### No change in final body weight with 8 weeks of wheel running

Baseline body weights were the same between groups (SED: 402·4 g (sd 11·2); EX: 394·1 g (sd 6·85); and SED-WM: 400·3 g (sd 9·11), *P* = 0·819). A main effect of time (*P* < 0·001, *F* (7182) = 435·86) and interaction effect of time × group (*P* < 0·001, *F* (14 182) = 3·33) were observed. No main effect of group was observed (*P* = 0·059, *F* (2,26) = 3·17) (Figure 1(b)). A follow-up ANOVA showed that body weight was different between groups at week 3 (*P* = 0·043, *F* (2,26) = 3·56), week 4 (*P* = 0·026, *F* (2,26) = 4·21), week 5 (*P* = 0·032, *F* (2,26) = 3·92) and week 6 (*P* = 0·045, *F* (2,26) = 3·52). EX had lower body weight than SED in week 3 (*P* = 0·037, *d =* 1·05), week 4 (*P* = 0·032, *d =* 1·06) and week 5 (*P* = 0·042, *d =* 1·00). No differences in body weight between groups were observed in week 1 (*P* = 0·316, *F* (2,26) = 1·20), week 2 (*P* = 0·127, *F* (2,26) = 2·24), week 7 (*P* = 0·064, *F* (2,26) = 3·06) or week 8 (*P* = 0·095, *F* (2,26) = 2·58).

### Exercise distance peaked at week 3

A main effect of time was observed (*P* < 0·001, *F* (7,63) = 11·91) (Figure 1(c)). Exercise distance was higher in all weeks relative to week 1 (*P* < 0·05). Exercise distances increased from weeks 1 to 2 (*P* = 0·005, *d =* 1·18) and weeks 2 to 3 (*P* = 0·005, *d =* 1·14). Exercise peaked at week 3 and stayed constant until week 6 when exercise decreased between weeks 6 and 7 (*P* = 0·012, *d =* 0·99). Total exercise distance was 192 (sd 110) km (range 56–332 km) (Figure 1(d)). In agreement with previous literature showing that ∼10 % of rats are non-runners^(46)^, one rat was excluded from EX due to lack of exercise.

### Energy intake was initially lower with exercise, but gradually increased with exercise training

**Figure 2.**
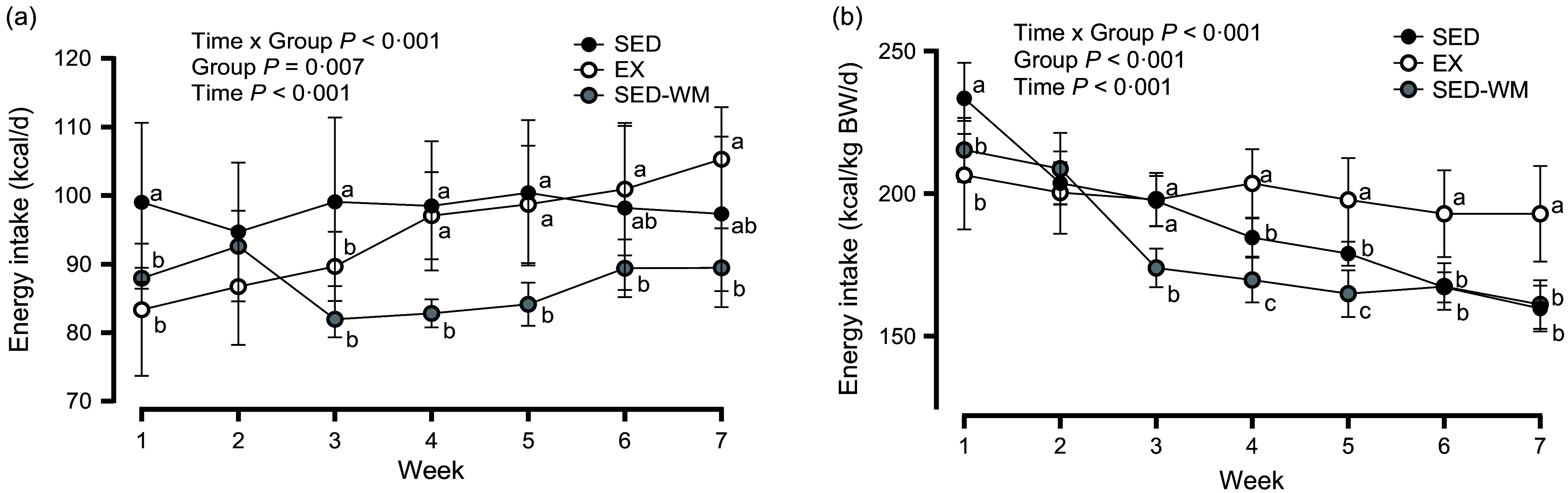
Weekly energy intake, expressed as kcal/d (a) and kcal/d per kg of body weight (b) for SED, EX and SED-WM from weeks 1 to 7. Values are means (sd). *n* 10 for SED and SED-WM; *n* 9 for EX. Week 8 energy intake data is not highlighted, as procedures from OGTT interfered with food intake. When a group × time effect was observed. ^abc^Superscripts indicate significant group differences, labelled means at a time without a common letter differ, *P* < 0·05. EX, exercise; SED, sedentary; SED-WM, sedentary - weight-matched.

To weight match SED-WM to EX group, food intake was restricted within the range of 83·8–97·8 % of SED with an average of 89·6 % throughout the duration of the study. A main effect of time (*P* < 0·001, *F* (6156) = 16·69) and time × group interaction (*P* < 0·001, *F* (12 156) = 19·59) was observed (Figure 2(a)). A follow-up ANOVA showed a difference in EI between groups at week 1 (*P* = 0·002, *F* (2,26) = 8·19), week 3 (*P* < 0·001, *F* (2,26) = 11·73), week 4 (*P* < 0·001, *F* (2,26) = 16·55), week 5 (*P* < 0·001, *F* (2,26) = 12·13), week 6 (*P* = 0·027, *F* (2,26) = 4·15) and week 7 (*P* = 0·002, *F* (2,26) = 8·11). An early reduction in EI in EX, relative to SED, was evident in week 1 (*P* = 0·002, *d =* 1·46) and week 3 (*P* = 0·040, *d =* 0·98). This was followed by a steady increase in EI in EX where EI was the same between EX and SED for the remainder of the study period. SED-WM had lower EI than SED in week 1 (*P* = 0·023, *d =* 1·33) and week 3–5 (*P* < 0·001, *d =* 1·93, 2·29, 2·08, respectively). SED-WM had lower EI than EX in week 4 (*P* < 0·001, *d =* 3·08), week 5 (*P* = 0·002, *d =* 2·31), week 6 (*P* = 0·029, *d =* 1·58) and week 7 (*P* = 0·001, *d =* 2·37). Time and group interacted to affect EI/kg BW (*P* < 0·001, *F* (12 156) = 19·88) (Figure 2(b)). Group differences were present at all time points, apart from week 2 (*P* < 0·001, *F* (2,26) = 1·23). Initially, at week 1, SED consumed more EI/kg BW than EX (*P* = 0·001, *d =* 1·69) and SED-WM (*P* = 0·025, *d =* 1·52). During weeks 2 and 3, EI/kg BW between EX and SED did not differ. After week 3, EI/kg BW remained greater in EX relative to SED (*P* < 0·001, *d =* 1·99, 1·79, 2·33, 2·58, respectively). EI/kg BW during week 7 was positively correlated with total exercise distance (*r* 0·829, *P* = 0·007). There was a positive correlation between total EI and body weight (*r* 0·824, *P* < 0·001). Week 8 EI data is not highlighted as fasting procedures for the OGTT interfered with food intake.

### Lower adiposity with exercise relative to sedentary rats

**Figure 3.**
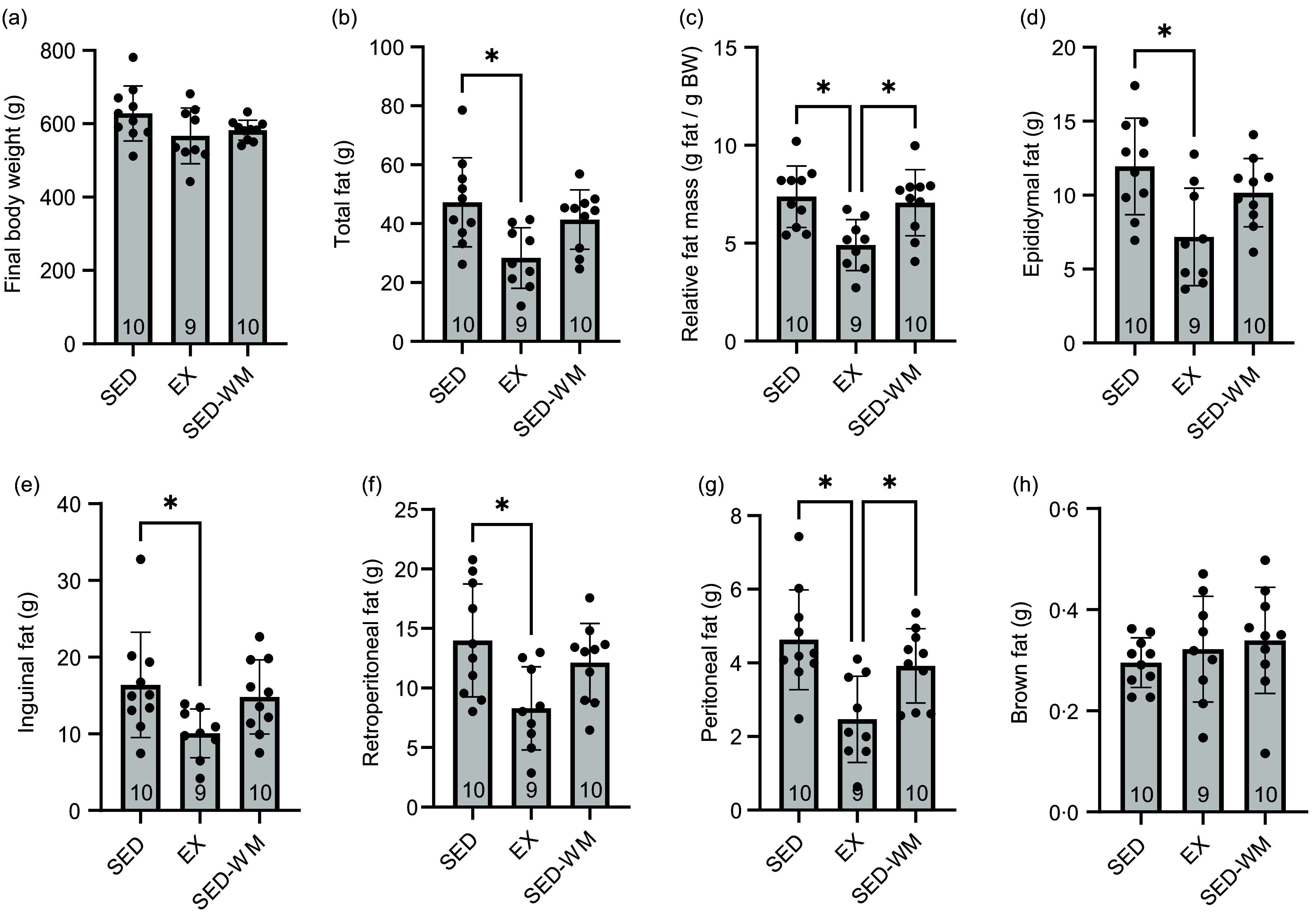
Body weight (a), total fat (b), relative fat mass (c), epididymal fat (d), inguinal fat (e), retroperitoneal fat (f), peritoneal fat (g) and brown fat (h) for SED, EX and SED-WM after 8 weeks. Values are means (sd). *n 9–*10/gp. *Significant group differences, Tukey’s post hoc comparison, *P* < 0·05. EX, exercise; SED, sedentary; SED-WM, sedentary - weight-matched.

A group difference was observed between epididymal (*P* = 0·007, *F* (2,26) = 6·148), retroperitoneal (*P* = 0·013, *F* (2,26) = 5·20), peritoneal (*P* = 0·002, *F* (2,26) = 8·051) and inguinal (*P* = 0·039, *F* (2,26) = 3·673) fat pad mass (Figure 3). A *post hoc* test showed that EX had reduced epidydimal (*P* = 0·005, *d =* 1·45), inguinal (*P* = 0·037, *d =* 1·16), retroperitoneal (*P* = 0·010, *d =* 1·36) and peritoneal (*P* = 0·001, *d =* 1·70) fat pad mass relative to sed. EX had lower peritoneal fat (*P* = 0·034, *d =* 1·33) relative to SED-WM. Overall, a difference was observed for total fat mass (*P* = 0·007, *F* (2,26) = 5·958) and relative fat mass (*P* = 0·003, *F* (2,26) = 7·166) between groups (Figure 3). A *post hoc* test showed that EX had lower total fat mass relative to SED (*P* = 0·006, *d =* 1·44) but not SED-WM (*P* = 0·068, *d =* 1·28). EX had lower relative fat mass compared to SED (*P* = 0·005, *d =* 1·71) and SED-WM (*P* = 0·013, *d =* 1·43).

### Measures of glycaemic control improved with exercise

#### Glucose

**Figure 4.**
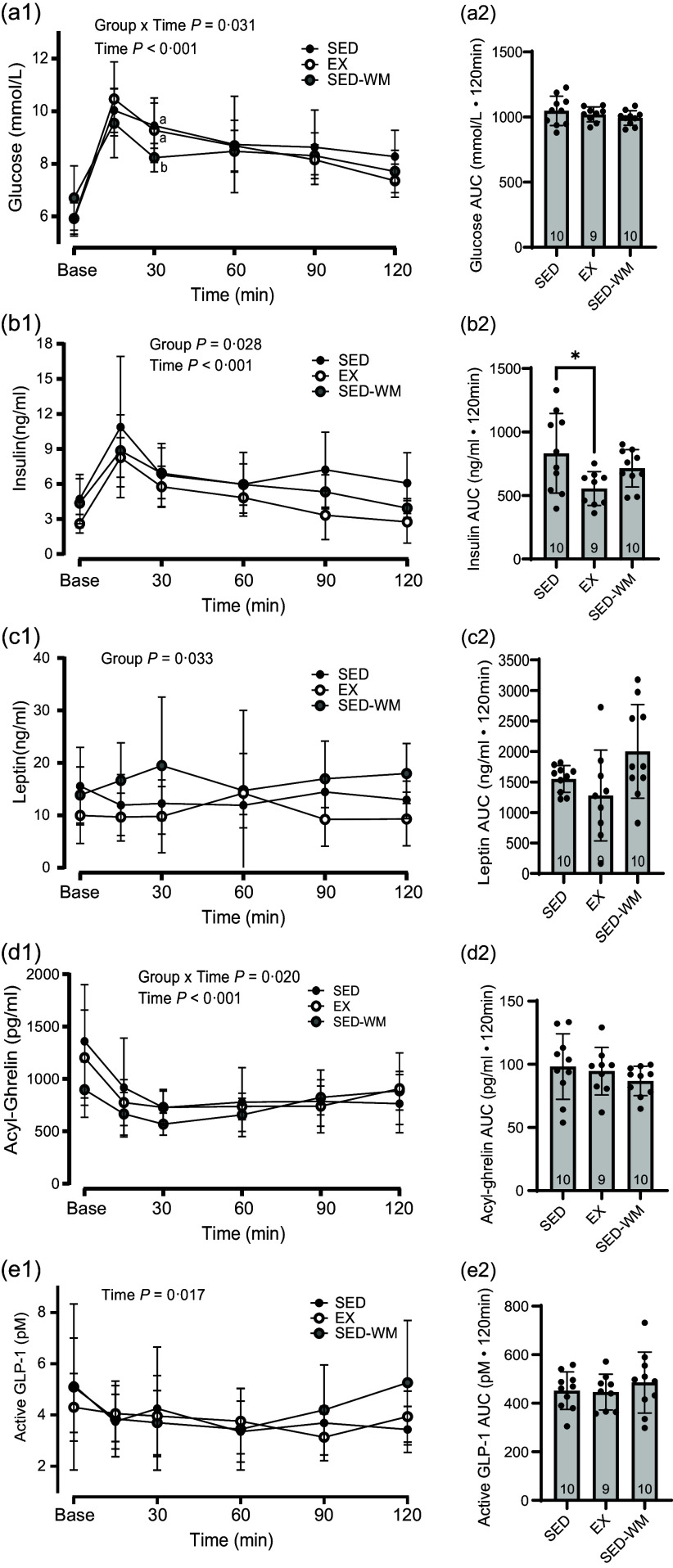
Glucose and appetite-regulating hormones. Concentrations (1) and AUC (2) for glucose (a), insulin (b), leptin (c), acyl ghrelin (d) and active GLP-1 (e) between SED, EX and SED-WM during a 120-minute oral glucose tolerance test. Values are means (sd). *n* 10 for SED and SED-WM; *n* 9 for EX. When a group × time effect was observed. ^ab^Superscripts indicate *significant group differences, labelled means at a time without a common letter differ, Tukey’s post hoc comparison, *P* < 0·05. EX, exercise; GLP-1, glucagon-like peptide-1; SED, sedentary; SED-WM, sedentary- weight-matched.

There was a time × group interaction effect (*P* = 0·031, *F* (10 130) = 2·08) and the main effect of time (*P* < 0·001, *F* (5130) = 52·73) for glycaemic response to an OGTT (Figure 4(a1)). No differences were observed between groups (*P* = 0·407, *F* (2,26) = 0·93). AUC glucose concentration did not differ between groups (Figure 4(a2)).

#### Insulin sensitivity

A main group effect was observed for homeostatic model assessment of insulin resistance (*P* = 0·036, *F* (2,26) = 3·79) and composite insulin sensitivity index score (*P* = 0·026, *F* (2,26) = 4·23) (Table 2). Homeostatic model assessment of inulin resistance was lower in EX relative to SED-WM (*P* = 0·048, *d =* 1·25). Composite insulin sensitivity index score was greater in EX in relation to SED, indicating improved insulin sensitivity in EX (*P* = 0·035, *d =* 1·23).

**Table 2.** Insulin sensitivity during an oral glucose tolerance test

	SED		EX		SED-WM	
	Mean	sd	Mean	sd	Mean	sd
HOMA-IR (au)	28·25	13·87^ab^	15·39	4·84^a^	29·45	14·78^b^
CISI (au)	1·68	0·69^a^	2·53	0·68^b^	1·76	0·72^ab^

au, arbitrary units; EX, exercise; CISI, composite insulin sensitivity score; HOMA-IR, homeostasis model assessment of insulin resistance; SED, sedentary; SED-WM, sedentary weight matched.

Values are means (sd). *n* 9–10/gp.

^ab^Superscripts indicate significant group differences, labelled means at a time without a common letter differ, Tukey’s post hoc comparison, *P* < 0·05.

### Tonic hormones insulin and leptin were both lowered with exercise training

#### Insulin

Baseline concentrations of insulin were different between groups at the start of the OGTT (*P* = 0·040, F (2,26) = 3·65), with SED having greater insulin than EX (*P* = 0·045, *d =* 1·29). A main effect of time (*P* < 0·001, *F* (5130) = 28·41) and group (*P* = 0·028, *F* (2,26) = 4·10) were observed (Figure 4(b1)). A *post hoc* test identified a group difference between EX and SED (*P* = 0·022, *η*_*p*_^*2*^ = 0·28), with EX having lower insulin relative to SED. A group difference in AUC insulin was observed (*P* = 0·033, *F* (2,26) = 3·89), with SED having higher AUC insulin than EX (*P* = 0·026, *d =* 1·13) (Figure 4(b2)). There was a positive correlation between AUC insulin and final body weight (*r* 0·473, *P* = 0·010), total fat (*r* 0·695, *P* < 0·001) and relative fat (*r* 0·673, *P* < 0·001). EI/kg BW during week 7 was negatively correlated with fasting OGTT insulin (*r* = –0·474, *P* = 0·009).

#### Leptin

**Figure 5.**
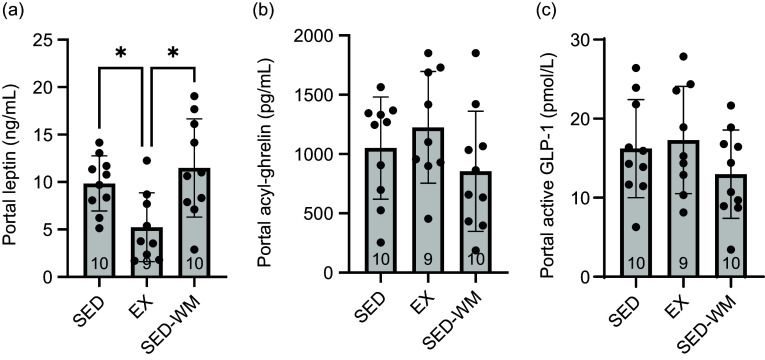
Fasting portal blood concentrations of appetite-regulating hormones in SED, EX and SED-WM after 8 weeks. Portal leptin (a), active GLP-1 (b) and acyl ghrelin (c). Values are means (sd). *n 9–*10/gp. *Significant group differences, Tukey’s post hoc comparison, *P* < 0·05. EX, exercise; SED, sedentary; SED-WM, sedentary weight-matched.

A main effect of group was observed (*P* = 0·033, *F* (2,26) = 3·91) (Figure 4(c1)). A *post hoc* test identified that EX had lower leptin relative to SED-WM (*P* = 0·026, *η*_*p*_^*2*^ = 0·25). Group differences in AUC leptin trended towards significance (*P* = 0·053, *F* (2,26) = 3·29) (Figure 4(c2)). A main effect of group on portal vein leptin concentration was observed (*P* = 0·007, *F* (2,26) = 6·03) (Figure 5(a)). A *post hoc* test showed a difference between EX *v*. SED (*P* = 0·049, *d =* 1·41) as well as EX *v*. SED-WM (*P* = 0·006, *d =* 1·38). There was a positive correlation between fasting leptin during the OGTT and portal leptin with final body weight (*r* 0·571, *P* = 0·001; *r* 0·440, *P* = 0·017, respectively), total fat (*r* 0·696, *P* < 0·001; *r* 0·736, *P* =< 0·001, respectively) and relative fat (*r* 0·666, *P* < 0·001; *r* 0·736, *P* < 0·001, respectively). EI/kg BW during week 7 was negatively correlated with portal leptin (*r* = –0·498, *P* = 0·006).

### Short-term appetite-regulating hormones were not affected by chronic exercise

#### Acylated ghrelin

A main effect for time (*P* < 0·001, *F* (5130) = 15·43) and an interaction effect between time × group (*P* = 0·020, *F* (10 130) = 2·23) were observed (Figure 4(d1)). No differences in AUC acylated ghrelin concentration (Figure 4(d2)) or portal acylated ghrelin concentration (Figure 5(b)) were observed between groups. Total exercise distance positively correlated with fasting acylated ghrelin (*r* 0·862, *P* = 0·003).

#### Active glucagon-like peptide-1

A main effect of time was observed (*P* = 0·017, *F* (5130) = 2·87) (Figure 4(e1)). Fasting and 15 min, post-glucose bolus active GLP-1 concentrations were the same. No differences in AUC GLP-1 (Figure 4(e2)) or portal GLP-1 concentration (Figure 5(c)) were observed between groups. Total exercise distance was negatively correlated with fasting GLP-1 (*r* = –0·671, *P* = 0·048).

#### Exercise lowers corticosterone

**Figure 6.**
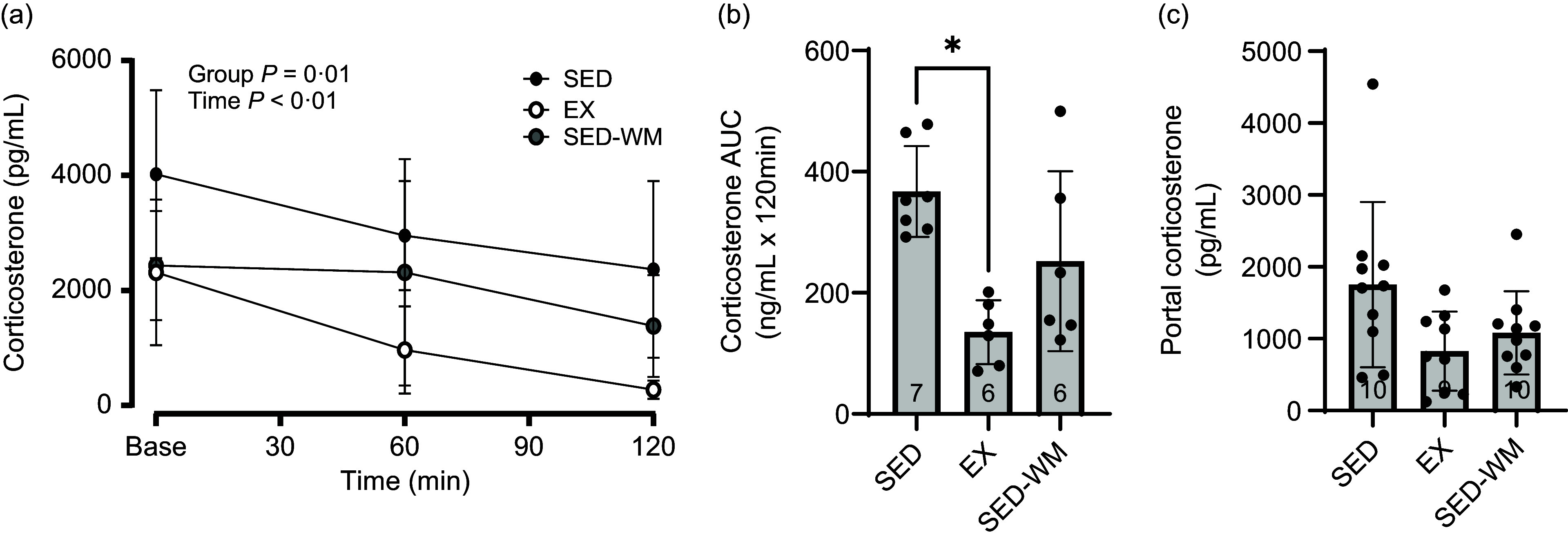
Measures of plasma corticosterone for SED, EX and SED-WM after 8 weeks. Concentration at time 0, 60 and 120 minutes during a 120-minute OGTT (a), total AUC during OGTT (b) and portal plasma (c). Values are means (sd). *n* 6–7/gp for (a) and (b); *n 9–*10/gp for (c). *Significant group differences, Tukey’s post hoc comparison, *P* < 0·05. EX, exercise; OGTT, oral glucose tolerance test; SED, sedentary; SED-WM, sedentary weight matched.

A main effect of time (*P* < 0·001, F (2,32) = 8·98) and group effect (*P =* 0·001, *F* (2,16) = 10·04) were observed (Figure 6(a)). A *post hoc* comparison demonstrated that EX had lower corticosterone than SED (*P* = 0·001, *η*_*p*_^*2*^ = 0·74). A group difference in AUC corticosterone concentration was observed (*P* = 0·003, F (2,16) = 8·86) (Figure 6(b)), with EX having lower AUC corticosterone relative to SED (*P* = 0·002, *d =* 3·53). No differences between groups were observed for portal corticosterone (*P* = 0·053, *F* (2,26) = 3·30) (Figure 6(c)). Sample size for Figure 6(a) and (b) was limited to *n* 6/gp given constraints with ELISA plates.

## Discussion

This study sought to understand the temporal impact of 8 weeks of voluntary wheel running on EI and the homeostatic, appetite-regulating hormones that influence appetite. Body weight was initially reduced in EX, but no differences were present between SED and EX at the end of the study. A reduction was observed in relative fat mass in EX compared with both SED and SED-WM. While exercise initially reduced EI, these short-term changes preceded a gradual increase in EI with continued exercise training to the point where EI, on a relative basis, was greater than SED. No differences in acylated ghrelin or GLP-1 were observed at the end of 8 weeks. Concomitant with the reduction in adiposity, EX reduced levels of long-term anorexigenic hormones leptin and insulin. Based on these findings, energy compensation observed with chronic exercise is influenced to a greater extent by long-term anorexigenic hormones than short-term, gut-derived appetite-regulating hormones.

All groups in our study progressively increased body weight over the course of 8 weeks. During the initial weeks of the study, body weight was lower with exercise. At the conclusion of 8 weeks of exercise, however, there were no differences in final body weight between groups. The pattern of EI over the 8 weeks followed a similar trajectory to that of body weight. Initially, EX had reduced EI relative to SED. This pattern shifted towards the mid-point of the study with EX slowly increasing EI. The reduction in EI with the onset of exercise has been observed previously^(47–49)^ and is consistent with the early anorexigenic effects observed with acute exercise^(13)^. As exercise exposure continues, there appears to be a gradual coupling of EE with EI^(50)^. Numerous animal studies using running wheels have reported elevations in EI with exercise training, with increases in EI ranging from ∼10 to 45 % with exercise durations of 5–10 weeks^(8,22,40,51)^. These increases in EI appear to be one factor contributing to similar body weights between exercise and sedentary groups. In our study, there was a positive correlation between EI/kg BW during week 7 and total exercise distance. This association has been reported previously^(22)^ and highlights the orexigenic potential of long-term exercise.

Although many studies show increases in EI with exercise, there are exceptions. Foright *et al.* reported reductions in EI and subsequent decreases in body weight with long-term exercise in male rats^(24)^. The reason for the discrepancy with our results is not entirely clear but may relate to a shorter exercise duration and the use of forced wheel exercise. Interestingly, despite reductions in EI with male rats, female rats responded to the same treadmill exercise protocol with increased EI and no change in body weight relative to sedentary animals. Greater energy compensation with voluntary wheel running in female animals has been reported previously^(52)^. Interestingly, female rodents also complete higher volumes of exercise relative to males^(53)^. The precise mechanisms mediating this differential appetite response between males and females remain unclear. It is speculated that the elevated EI observed in female rats with exercise may be related to the need to preserve body tissue and protect reproductive function^(52)^. The influence of appetite-regulating hormones on the sex-specific appetite response remains poorly understood. Further investigation is required to examine how sex differences in appetite-regulating hormones impact energy compensation with chronic exercise.

Another factor that may influence outcomes related to EI with exercise is diet and associated metabolic state. Animals fed a high-fat diet, relative to a low-fat diet, commonly respond to an exercise intervention with reduced body weight and a reduction in EI^(23,54,55)^. This has been reported in 4 week^(54)^, 5 week^(55)^ 6 week^(40,48)^, 8 week^(56)^, 10 week^(57)^, 12 week^(23,25)^ and 40 week^(58)^ studies. The reason for a greater reduction in the high-fat diet relative to a lean diet may be related to altered food-reward appetite signalling. Both exercise and highly palatable diets stimulate the reward centres in the brain^(55)^. Through activation of hedonic reward centres, exercise may reduce the preference for highly palatable diets^(55,59)^. Mechanisms underpinning this central reward replacement are purported to include enhanced leptin signalling in the ventral tegmental area^(60)^ and/or changes in gene expression in the mesolimbic reward pathway^(59)^. The AIN-93M diet used in our study was a lean diet, with fat content around 10 %. With the utilisation of a lean diet, it is possible that food reward did not play a prominent role in eating behaviour, thus allowing the homeostatic influence of exercise on EI to emerge.

Although there is some uncertainty regarding the impact of exercise on body weight, most studies demonstrate that exercise improves body composition^(7,26,27,40,48)^. In agreement with these studies, we found that total fat pad mass and relative fat mass were reduced by ∼40 % and ∼33 %, respectively, with exercise. Epididymal, inguinal, retroperitoneal and peritoneal fat pads were reduced in EX compared with SED. Highlighting the exercise-dependent nature of the reduction in adiposity, our findings show that EX had a lower relative fat mass than SED-WM. Not all studies report reductions in body fat with exercise training. Jung *et al.* found no statistical differences in body composition with exercise^(61)^. As noted by the authors, one possible explanation for this finding was a reduction in non-wheel activity within the cage in the exercise group. Although we were unable to assess the potential contribution of non-exercise activity thermogenesis to energy compensation in our study, physical activity patterns were sufficient to induce the commonly observed changes in body composition, even with exercise volumes diminishing towards the latter part of the study. Despite differences in fat pad mass, no differences were observed in brown adipose tissue weight between groups. The maintenance of brown adipose tissue in EX may relate to the sympathetic nervous system stimulation that accompanies exercise along with other exercise-related factors^(62)^.

Alongside the reduction in adiposity, our results show that exercise reduced leptin and insulin. The reductions of these appetite-influencing hormones occurred in a weight-independent manner, as indicated by the maintained concentrations of these hormones in the SED-WM group. Fasting and postprandial leptin^(33,39,63)^ and insulin^(23,37,40)^ concentrations have been observed to decrease after chronic exercise. The reduction in these homeostatic hormones is one explanation for the elevated EI observed with habitual exercise. In our study, a negative association between leptin and insulin was observed with EI/kg BW. Although we did not serially measure leptin concentrations in our study, exercise studies in rodents demonstrate a steady decline in leptin concentrations with time^(48)^. With the gradual reductions in body fat and subsequent reduction in leptin with exercise, the decrease in leptin is a likely factor contributing to the gradual coupling of physical activity with EI. Despite this purported association between reduced leptin and increased EI, not all studies support this link. Foright *et al.* explored the impact of 4 weeks of forced treadmill exercise on body weight and EI in rats. Despite reductions in leptin in male rats with exercise, the reduction in leptin did not influence EI. Using a leptin responsiveness test, it was determined that exercise did not impact the anorectic effects of leptin^(24)^. Furthermore, a human exercise trial demonstrated that a reduction in leptin AUC was the only independent predictor of reduced energy compensation with exercise^(64)^. In the absence of leptin being responsible for exercise-mediated increases in EI, it is not clear what physiological factors may be responsible for the couple of EE with EI. It is hypothesised that EI may be driven by fat-free mass^(65)^. As lean tissue increases with exercise training, it is plausible that this drives EI. One limitation with this current study is that we did not assess fat-free mass. With future research, it would be important to accurately assess lean tissue and examine associations between lean tissue and EI.

To date, the impact of chronic exercise on episodic appetite hormones, independent of weight change, has not been thoroughly assessed. In response to 8 weeks of exercise training or energy restriction, we observed no differences in GLP-1 or acyl ghrelin in response to an OGTT. Furthermore, fasting portal vein concentrations of these hormones were the same between groups. Previous studies assessing chronic exercise in rodents have demonstrated anorexigenic changes in episodic hormones with elevations in peptide YY^(23,66)^ and reductions in acylated ghrelin^(23,37)^. In contrast to these findings, rodent studies have also highlighted elevations in ghrelin with wheel running^(45)^. While noting that findings are mixed regarding the impact of chronic exercise on acyl ghrelin, a review conducted by Ouerghi *et al.* of exercise-based studies in humans suggests that acyl ghrelin concentrations are generally increased in response to chronic exercise^(67)^. Alongside increases in acyl ghrelin, human research also shows reductions in GLP-1 and peptide YY with 12 weeks of supervised exercise training^(68)^. In keeping with these findings, we identified a negative correlation between total exercise distance and GLP-1 and a positive correlation between exercise distance and acylated ghrelin. These associations demonstrate a physiological defence to an exercise-induced energy deficit. Given that greater energy compensation through EI has been observed in female animals^(24,52)^, future work should examine the potential role of these short-term appetite hormones in mediating this response.

An interesting finding from our study was the heightened baseline concentration of GLP-1 relative to the GLP-1 concentration measured 15-min post-glucose gavage. As is typically observed with GLP-1 measures, plasma GLP-1 increases after a glucose bolus or meal^(69)^. In our study, we observed unchanged concentrations of GLP-1. We speculate that this was related to the stress–response induced by the tail vein catheterisation protocol used in our study. To examine whether this was the case, we measured the concentration of corticosterone at baseline, 60 min and 120 min post-OGTT. Corticosterone concentrations were indeed elevated at baseline. With acute exercise, one of the purported mechanisms for the transient increase in GLP-1 is acute sympathetic response^(70)^. Based on the elevation of active GLP-1 with the tail vein catheterisation, it is likely that an acute stress–response triggered this early GLP-1 release. For the purpose of this study, it remains unclear whether this stress–response masked potential differences in baseline measures of appetite-regulating hormones due to exercise training.

### Conclusion

In conclusion, our results support previous research showing that voluntary exercise, despite inducing early reduction in body weight, does not contribute to long-term weight change. Incremental increases in EI with exercise exposure serve to negate reductions in body weight over time. Appetite-regulating hormones may play a role in mediating this response. Alongside reductions in adiposity with exercise, the decrease in long-term anorexigenic hormones leptin and insulin are likely to contribute to the orexigenic response associated with exercise. Despite the potential for exercise to influence short-term, gut-derived appetite-regulating hormones, these hormones do not appear to play a prominent role in energy compensation with voluntary exercise.
